# The effectiveness of olfactory training for chronic olfactory disorder following COVID-19: a systematic review

**DOI:** 10.3389/fnhum.2024.1457527

**Published:** 2024-11-11

**Authors:** Natalia Treder-Rochna, Aleksandra Mańkowska, Wiktoria Kujawa, Michał Harciarek

**Affiliations:** Faculty of Social Sciences, Institute of Psychology, University of Gdansk, Gdansk, Poland

**Keywords:** chronic olfactory disorder, COVID-19, olfactory training, smell, adjuvants

## Abstract

**Background:**

Chronic olfactory disorders are some of the most frequent post-COVID-19 presentations. Olfactory training (OT) is currently the most popular method used for treating post-viral olfactory dysfunction (PVOD). We evaluated the effect of olfactory training on the chronic olfactory disorders of patients infected with COVID-19.

**Methodology:**

A systematic literature search was performed per PRISMA guidelines in PubMed, Scopus, Web of Science, EBSCOhost, and the Cochrane Library. Only patients with chronic olfactory disorders of 30 days or more were included. The primary outcome was the olfactory score at the end of follow-up. In all studies, improvement was defined as a positive change over time in the results of objective psychophysical olfactory tests. The most commonly used test was the Sniffin' Sticks. Typically, outcome measures involved comparing the mean olfactory scores. In the Sniffin' Sticks test, an improvement was also indicated by a change of 5.5 points or more in the Threshold, Discrimination, and Identification scores.

**Results:**

Fourteen studies (1.596 participants) were included in this review. Among the included studies, up to 10 were RCTs. Nine studies assessed the combined effects of adjuvant therapy and olfactory training, while five studies assessed only OT.

**Conclusions:**

In our assessment, olfactory training alone produces significant improvements in chronic olfactory dysfunctions. However, a combined therapy approach is essential to achieve more effective outcomes. Integrating olfactory training with adjuvants like CoUltraPEALut, Cerebrolysin, and oral Vitamin A has demonstrated substantial benefits in enhancing post-COVID-19 olfactory function. Strict adherence to the OT protocol and extending the duration of OT to 3 months or more significantly enhance treatment outcomes.

## 1 Introduction

Olfactory dysfunction (OD) is one of the most prevalent symptoms of long COVID, which, according to the World Health Organization (WHO, 2021), is defined as the presence of signs and/or symptoms that persist for at least 2 months after 3 months from the initial infection, and cannot be explained by other medical conditions. Most individuals regain their sense of smell within a month, and 15%−46% experience persistent OD (Boscolo-Rizzo et al., 2021; Ferreli et al., 2022). Currently, there are no established guidelines for treating COVID-19-related chronic olfactory disorders. However, some meta-analyses (Asvapoositkul et al., 2023; Hwang et al., 2023), as well as the systematic review conducted by Hwang et al. (2022), suggest that olfactory training may improve olfactory function in post-COVID-19 patients. Nevertheless, a few studies have reported that OT may not provide benefits for COVID-19-related olfactory dysfunction (Di Stadio et al., 2022; Hamed and Ahmed, 2023; Cantone et al., 2024). Similarly, Bérubé et al. (2023) found no effect of OT on objective olfactory test scores, though significant improvements in subjective olfactory function and reduced parosmia were noted in the intervention group. Some researchers emphasize that while they do observe an effect of OT on olfactory dysfunction in objective assessments, they describe these effects as modest (Le Bon et al., 2021; Fjaeldstad et al., 2023).

The mechanism of olfactory training is not entirely understood. It is believed to involve neuroplasticity—the brain's ability to modify its structure and function in response to sensory input and experience. Neuroplasticity is a crucial characteristic of the olfactory system, which exhibits a capacity for regeneration (Schwob, 2002; Kim et al., 2019). Studies using animal models have shown that olfactory training enhances the connections between the olfactory bulb and key brain regions involved in olfactory perception, such as the piriform cortex and the orbitofrontal cortex (Courtiol and Wilson, 2017). This enhanced connectivity can potentially improve odor processing and heighten the perception of smell. Neuroimaging studies have shown that olfactory training is associated with both structural and functional changes in the human brain (Li et al., 2006; Kollndorfer et al., 2015; Negoias et al., 2017; Hosseini et al., 2020), emphasizing its ability to modify neural pathways involved in olfactory processing. Moreover, OT has been considered an appealing therapeutic option due to its simplicity in administration, affordability, and minimal side effects.

Previous systematic reviews and meta-analyses suggest that olfactory training may be an effective intervention for patients with olfactory dysfunction of various etiologies and durations, such as post-infectious, post-traumatic, and chronic inflammatory conditions (e.g., rhinosinusitis, rhinitis) (Pekala et al., 2016; Sorokowska et al., 2017; Huang et al., 2021; Kattar et al., 2021). The meta-analysis by Kattar et al. (2021) further found that, in cases of persistent olfactory disorders, a longer duration of olfactory training is associated with greater improvements in olfactory function, while a longer duration of symptoms is linked to worse outcomes. However, the meta-analysis by Asvapoositkul et al. (2023) did not support these findings, showing that olfactory training was effective in five studies involving post-COVID-19 patients, with three of these studies involving patients with olfactory dysfunction lasting more than 30 days (D'Ascanio et al., 2021; Denis et al., 2021; Saussez et al., 2021). Similarly, Hwang et al. (2023) found that olfactory training improved olfactory scores in patients with both acute and chronic olfactory dysfunctions related to COVID-19.

This review is not the first to analyze the use of olfactory training in COVID-19 patients (Hwang et al., 2022, 2023; Veronese et al., 2022; Asvapoositkul et al., 2023; Wang et al., 2023). However, previous reviews summarized studies that focused on various therapy methods, treating olfactory training as one of several interventions (Veronese et al., 2022; Asvapoositkul et al., 2023; Wang et al., 2023). In connection with this, a limited number of studies specifically focused on olfactory training were included in the analyses. In the review by Asvapoositkul et al. (2023), five studies were included (one was excluded due to incomplete data for analysis), while Wang et al. (2023) included three studies on olfactory training; however, they did not analyze these studies in terms of the effectiveness of the olfactory training itself. In contrast, Veronese et al. (2022) included only one study on OT. Persistent long-term olfactory disorders were only minimally considered and did not form the main subject of analysis (Asvapoositkul et al., 2023; Wang et al., 2023). Moreover, one review excluded studies involving olfactory dysfunction lasting 30 days or longer after COVID-19 (Hwang et al., 2022). In contrast, Hwang et al. (2023) included six studies examining prolonged olfactory issues in long COVID cases. However, the researchers did not apply rigorous inclusion and exclusion criteria, such as omitting patients with olfactory problems stemming from head injuries, chronic neurodegenerative conditions, pre-COVID olfactory disorders, or severe nasal diseases. Therapeutic options for patients with long COVID were the subject of analysis in the review by Veronese et al. (2022). However, the researchers included only one study concerning OT in patients with chronic olfactory disorders. Given these limitations, there is a need for a dedicated systematic review focused on the effectiveness of OT (alone or in conjunction with other therapies) in long COVID patients with chronic olfactory disorders to provide clearer insights into this therapeutic approach. Moreover, research indicates that most patients experience spontaneous remission of olfactory dysfunction (Orji et al., 2023); however, it is crucial to address whether patients with persistent COVID-related olfactory dysfunctions associated with long COVID also benefit from olfactory training. Currently, there is also a significant gap in understanding the potential enhanced effects of combining OT with other treatments (Veronese et al., 2022; Asvapoositkul et al., 2023; Hwang et al., 2023; Wang et al., 2023). This systematic review aims to assess the effectiveness of olfactory training for chronic olfactory disorder persisting for 30 days or longer after COVID-19. Additionally, we evaluate the outcomes of olfactory training alone and olfactory training with adjuvants, including both pharmacological interventions and nutritional supplementation.

## 2 Materials and methods

### 2.1 Information sources and search strategies

We followed the PRISMA guidelines for reporting systematic reviews (Sarkis-Onofre et al., 2021). We searched PubMed, Scopus, Web of Science, EBSCOhost, and the Cochrane Library through 01 May 2024, for articles written in English. The search for individual studies was complemented by manually reviewing the reference lists of relevant systematic reviews that had already been published on this topic. The following search terms were used: (“COVID-19” OR “SARS-CoV-2” OR “Long COVID”) AND (“olfactory dysfunction” OR “anosmia”) AND (“olfactory training” OR “smell therapy”).

### 2.2 Research question

The research question for this systematic review is: Is olfactory training effective for chronic olfactory disorder persisting for 30 days or longer after COVID-19? This review will focus on several key aspects. First, it will evaluate whether the duration of olfactory training influences the recovery of olfactory function. The types of scent exposures used during the training will also be explored to determine their impact on olfactory recovery. Furthermore, the review aims to identify if certain patient subgroups benefit more significantly from olfactory training compared to others. This could indicate a need for tailored therapeutic approaches to maximize the effectiveness of the intervention. Additionally, the review will compare the outcomes of olfactory training alone with those of olfactory training combined with adjuvants, including both pharmacological interventions and nutritional supplementation. This comparison will help to establish whether combining olfactory training with other treatments provides additional benefits in the recovery of olfactory function in patients with chronic olfactory disorder after COVID-19. The research question was framed using the PICO(S) format (Table 1).

**Table 1:** PICO(S) components for systematic review.

**Component**	**Description**
Participants	Patients with COVID-19-related chronic olfactory dysfunction (chronic olfactory disorder persisting for 30 days or longer).
Intervention	Olfactory training
Comparison	Standard care, placebo, or no intervention.
Outcome	Improvement in olfactory function (subjective or objective olfactory assessment).
Study Design	Randomized controlled trials, case-controlled studies, cohort studies.

### 2.3 Inclusion and exclusion criteria

Inclusion criteria encompassed randomized controlled trials (RCTs), case-controlled studies, and cohort studies on olfactory training related to COVID-19.

Studies with an unclear follow-up, observational studies, and head-to-head trials were excluded. Non-peer-reviewed articles, abstracts, conference papers, and articles not available in English were omitted. Additionally, studies with significant methodological flaws were eliminated.

The population consisted of patients (≥18 years old) experiencing olfactory loss following a SARS-CoV-2 infection and who underwent olfactory training. The study covered patients with chronic olfactory disorder persisting for 30 days or longer. The average duration of olfactory dysfunction after COVID-19 was highly variable, but generally ranged from a few months to about a year. This criterion was used to minimize the number of patients who might show spontaneous recovery, regardless of the therapy. The population covered both male and female patients across different age groups. Patients had a confirmed history of COVID-19 infection with either RT-PCR or immunological testing.

Patients were excluded from the study if they were suffering from olfactory dysfunction due to other causes such as a previous history of head trauma, chronic neurodegenerative disease, or traumatic brain injury. Additionally, patients with a history of olfactory disorder prior to COVID-19 infection, severe sinonasal diseases, previous sinonasal surgery, or nasal cavity polyps were excluded. Dependence on prolonged corticosteroid therapy for comorbid conditions, such as asthma and chronic obstructive pulmonary disease, was also a criterion for exclusion.

The training could be supported by pharmacological interventions as well as appropriate supplementation. Studies were included that involved olfactory training supported by pharmacological interventions, such as topical and oral corticosteroids, as well as antioxidants like alpha-lipoic acid (ALA), mineral supplements that contained Palmitoylethanolamide (PEA), and Luteolin. Also included were studies involving supplementation, such as vitamin A, other vitamins, nutritional supplements, multivitamin B, or a combination of any of the above. The most commonly used therapies for OD in otolaryngology were considered, focusing solely on pharmacological interventions and supplementation. Interventions that did not include olfactory training (e.g., pharmacological treatments without an olfactory training component) were not considered.

The primary outcome of this study was to evaluate the change in olfactory scores following olfactory training. The outcomes were either subjective olfactory assessment: Visual Analog Score (VAS), Self-Rating Olfactory Score, or the Sino-Nasal Outcome Test (SNOT-22) (Hopkins et al., 2009) or objective olfactory assessment such as Sniffin' Sticks test [odor threshold (T), discrimination (D), or identification (I)], Sniffin' Sticks identification test (Hummel et al., 1997), University of Pennsylvania Smell Identification Test (UPSIT) (Doty et al., 1984), and Connecticut Chemosensory Clinical Research Center test (CCCRC) (Cain et al., 1988).

### 2.4 Study selection

**Figure 1:**
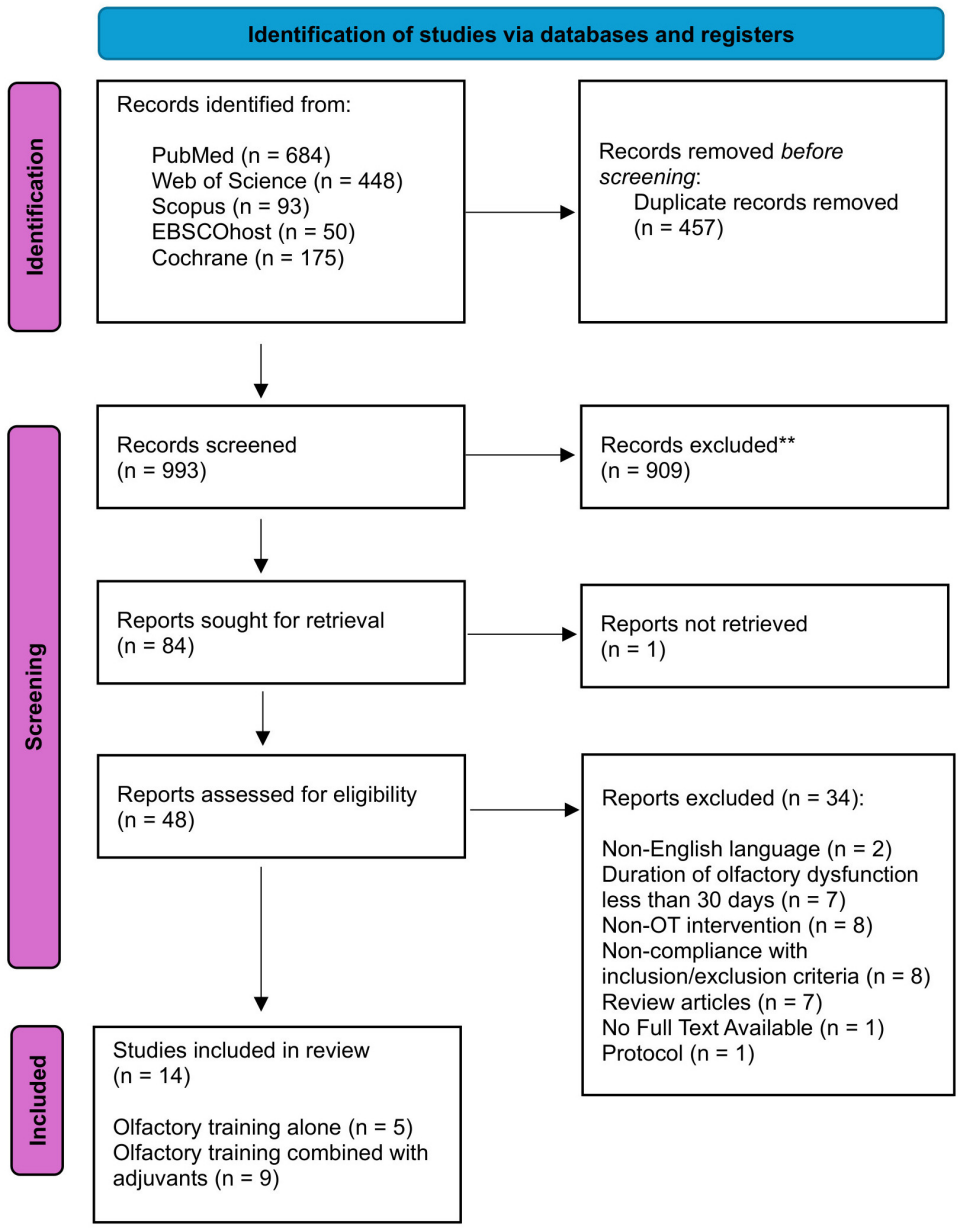
PRISMA diagram.

The study selection was independently conducted by two authors (NTR, AM), with consensus meetings held to resolve any discrepancies in their decisions. When necessary, a third member of the review team was consulted. The selection process began with an initial screening based on titles and/or abstracts, followed by a detailed evaluation of the full-text manuscripts of the studies identified in the first step. Research steps are shown in the PRISMA diagram (Figure 1).

### 2.5 Data extraction

Two authors independently extracted the following information:

Manuscript Details: First author name and affiliation, year of publication, journal name, manuscript title.

Population Characteristics: Sample size, mean age, gender, comorbidities, chronic olfactory disorders persisting for 30 days or longer, long COVID (according to WHO criteria), method of COVID-19 diagnosis, follow-up duration (in months), and inclusion and exclusion criteria.

Olfactory Dysfunction: Duration of dysfunction, assessment type (subjective vs. objective).

Olfactory Training Details: Treatment regimen (type of odor used - traditional odors by Hummel et al., 2009 or others), frequency, mean duration of training, follow-up duration, quality of odors.

Adjuvant Therapy: Use of additional therapies (yes/no/not reported), type of therapy.

Outcome Details: Primary outcomes were objective or subjective olfactory assessments.

## 3 Results

Fourteen articles involving 1.596 individuals were included from retrieved studies. 32% of the participants were male, and 68% were female. The mean age of patients in 12 out of 14 studies was 39.19 years. The median age for the remaining 2 studies was 44 and 49 years, respectively. Among the included studies, up to 10 were RCT. Nine studies assessed the combined effects of adjuvant therapy and olfactory training, while 5 studies assessed only OT. The study characteristics are shown in Table 2.

**Table 2:** Characteristics of included studies.

**Study (year), country**	**Study type**	**Sample size**	**Age (mean or median)/sex (M/F)**	**Long Covid**	**Duration of olfactory dysfunction (mean or median ±SD)**	**Intervention**	**Olfactory Training**	**Follow-up**	**Outcome measure analyzed**	**Effect Sizes** **(Cohen's *d)***
Bérubé et al., 2023, Canada	Double-blind randomized pilot study	Intervention: 50 Follow-up: 40	M = 44.7/17/33 (intervention)	Yes	approx. 8.44 months **Intervention group** M = 273 ± 108 days **Placebo group** M = 241 ± 110 days	(1) Intervention group: OT (2) Placebo group: odorless substances, unaware that the intervention involved smelling odors	4 scents: Rose, Orange, Eucalyptus, Clove, for 5 min, twice daily	3 months	**Objective:** University of Pennsylvania Smell Identification Test (UPSIT-40). **Self-reporting score:** Visual Analog Scale (VAS). Assessed the presence of parosmia.	**Intervention group**: d = 0.200; *p >* 0.05 **Placebo group:** d = 0.170; *p >* 0.05
Cantone et al., 2024, Italy	RCT	Intervention: 120 Follow-up: 89	M = 43.7/38/51 (follow-up)	Yes	approx. 10 months **UmPEALUT group** M = 10 ± 4.4 months **ALA group** M = 10.2 ± 4.13 months **Combined group** M = 15.3 ± 6.6 months **Control group** M = 8.1 ± 1.8 months	(1) UmPEALUT group: ultramicronized palmitoylethanolamide and luteolin + OT (2) ALA group: alpha-lipoic acid + OT (3) Combined group: umPEALUT + ALA + OT (4) Control group: OT	4 scents: Rose, Lemon, Eucalyptus, Cloves, for 6 min, three times per day	6 months	**Objective:** Sniffin' Sticks test (threshold, detection, and identification, TDI). **Self-reporting score:** Parosmia questionnaire.	**UmPEALUT group:** d = 1.220; *p < * 0.01 **ALA group:** d = 0.481; *p >* 0.05 **Combined group:** d = 1.650; *p < * 0.01 **Control group:** d = 0.155; *p >* 0.05
Chung et al., 2023, China	Randomized- controlled pilot study	Intervention: 26 Follow-up: 22	Md = 44/8/14 (follow-up)	Yes	approx. 5.22 months **Combination group** Md = 159 (130.0–163.0) days **Standard care group** Md = 164.5 (118.3–180.3) days **Control group** Md = 138.0 (135.0–225.0) days	(1) Combination group: oral vitamin A + OT (2) Standard care group: OT (3) Control group: clinical observation	4 scents: Lemon, Eucalyptus, Geranium, Cedarwood, three times per day	1 month	**Objective:** Olfactory function by butanol threshold tests (BTT), UPSIT, MRI assessments.	no date
de Sousa et al., 2023, Portugal	Comparative study	Intervention: 47 Follow-up: NI	M = 37.2/17/30 (intervention)	Yes	approx. 7.61 months (M = 231.7 ± 114.5 days)	(1) Nosal Topical Corticosteroid + OT (2) Nosal Topical Corticosteroid + Vitamin B complex + OT (3) Intranasal Vitamin A and E + OT (4) OT	4 scents: Rose, Lemon, Eucalyptus, Cloves, NI, three times per day	3 months	**Objective:** Sniffin' Sticks threshold test. **Self-reporting score:** VAS.	**All groups** d = 1.619; *p < * 0.01
Di Stadio et al., 2022, Italy	RCT	Intervention: 185 Follow-up: 185 (all completed the entire course of treatment)	M = 43.5/64/121	Yes	approx. 8.4 months **Intervention Group** M = 8.2 ± 3 months **Control Group** M = 8.8 ± 2.4 months	(1) Intervention Group: PEA-LUT + OT (2) Control Group: placebo (multivitamin, vitamin D, and/or alpha-lipoic acid, based on an evidence-based literature review showing that these doses do not exert significant systemic anti-inflammatory or immunomodulatory effects) + OT	4 scents: Rose, Lemon, Eucalyptus, Cloves, for 6 min, three times per day	3 months (patients in the intervention group were evaluated every 30 days, while the control group was re-evaluated only after 90 days).	**Objective**: Sniffin' Sticks test (TDI).	**Intervention Group** d = 1.194; *p < * 0.001 **Control Group** d = 0.171; *p >* 0.05
Figueiredo et al., 2024, Brazil	RCT	Intervention: 128 Follow-up: 100	M = 39.05/18/82 (follow-up)	Yes	approx. 7.5 months **Intervention group** Md = 8 (5–12) months **Placebo group** Md = 7 (5–10.5) months	(1) Intervention group: alpha-lipoic acid + OT (2) Placebo group: starch pills + OT	4 scents: Rose, Lemon, Eucalyptus, Cloves, twice daily	3 months	**Objective:** Connecticut Chemosensory Clinical Research Center test (CCCRC, threshold, detection, and identification). **Self-reporting score:** VAS.	**Intervention group** d = 1.353; *p < * 0.001 **Placebo group** d = 0.931; *p < * 0.001
Hamed and Ahmed, 2023, Egypt	RCT	Intervention: 250 Follow-up: 202	M = 31.3/93/157 (intervention)	Yes	approx. 11.7 months **Group 1** M = 11.6 ± 3.7 months **Group 2** M = 11.8 ± 3.7 months	(1) Group 1: cerebrolysin therapy + OT + gustatory training (GT) (2) Group 2 (controls): OT+GT	4 scents: e.g. Lemon, Curry Powder, Lavender, Pungent Herbs, Spices Cloves, and essential oils containing scents, for 6 min, at least two times a day. The training period was tailored to each patient's response and set to be at least 8 weeks, up to a maximum of 24 weeks.	3 and 6 months of discontinuation of interventions to determine whether or not there was maintenance of the same degree of recovery.	**Objective:** Sniffin' odor identification test (SOIT) (16 odorants familiar to Egyptians). **Self-reporting score:** Global Rating Scale of Smell (GRS).	no date
Khan et al., 2023, USA	RCT	Intervention: 275 Follow-up: 170	M = 41/39/236 (intervention)	Yes	approx. 6 months **Patient preferred (Bimodal)** M = 6 ± 3 months **Physician assigned (Bimodal)** M = 5 ± 3 months **Patient preferred (Unimodal)** M = 6 ± 3 months **Physician assigned (Unimodal)** M = 6 ± 3 months **Control** M = 5 ± 3 months	(1) Bimodal training with patient-preferred scents (2) Bimodal training with physician-assigned scents (3) Unimodal training with patient-preferred scents (4) Unimodal training with physician-assigned scents (5) Control group	Physician-assigned scents: 4 scents: Rose, Lemon, Eucalyptus, Clove. Patient-preferred scents: choice of 4 out of 24 available scents. Bimodal (visual-olfactory) arm: viewing digital images of the essential oil being smelled. All: twice daily.	3 months	**Objective:** UPSIT. **Self-reporting score:** Clinical Global Impressions (CGI) Severity and Improvement, Olfactory Dysfunction Outcomes Rating (ODOR).	**Patient preferred (Bimodal)** d = 0.511 **Physician assigned (Bimodal)** d = 0.430 **Patient preferred (Unimodal)** d = 0.261 **Physician assigned (Unimodal)** d = 0.328
Lechien et al., 2023, Belgium	Comparative study	Intervention: 97 Follow-up: 57	M = 40.55/23/34 (follow-up)	Yes	NI, 3-month post-COVID-19	(1) Full- adherence to OT (2) Non- adherence to OT	4 scents: Rose, Lemon, Eucalyptus, Cloves, twice daily.	6, 12, and 18 months	**Objective:** Sniffin' Sticks identification test. **Self-reporting score:** French version of the short version of Questionnaire of Olfactory Disorders-Negative Statements (sQOD-NS).	**Full- adherence to OT 6 months:** d = 0.864; *p* < 0.001 **Non- adherence to OT 6 months:** d = 0.950; *p* < 0.01
Pires et al., 2022, Brazil	RCT	Intervention: 80 Follow-up: 80	M = 36.7/28/52	No	approx. 2 months (M = 63.9 ± 24.2 days)	(1) Classical olfactory training (COT): 4 essential oils (2) Advanced olfactory training (AOT): 8 essential oils	COT: 4 scents: Rose, Lemon, Eucalyptus, Cloves, NI, twice daily. AOT: 8 scents: Rose, Eucalyptus, Clove, Lemon, Citronella, Mint, Vanilla, and Cedar wood, twice daily.	1 month	**Objective:** UPSIT. **Self-reporting score:** VAS.	**COT** **+** **AOT** d = 0.223; *p* < 0.05
Saussez et al., 2021, Belgium, Italy, Spain	Prospective observational controlled study	Intervention: 152 Follow-up: 152	M = 41.2/62/90	No	NI	(1) OC (10 days of oral corticosteroids) + OT (2) NC (1 month of nasal corticosteroids) + OT. (3) OT.	>3 daily odors: e.g., Coffee, Perfume, Essential Oils, twice daily.	1 and 2 months	**Objective:** Sniffin' Sticks identification test. **Self-reporting score:** National Health and Nutrition Examination Survey	no data
Schepens et al., 2022, Netherlands	RCT	Intervention: 115 Follow-up: 113	Md:49/42/73 (intervention)	Yes	loss of smell > 4 weeks	(1) Prednisolone group: prednisolone + OT (2) Placebo group: matching placebo + OT	NI, twice daily.	3 months	**Objective**: Sniffin' Sticks test (TDI). **Self-reporting score:** Sino-Nasal Outcome Test-22 questionnaire (SNOT-22), VAS, Olfactory Disorders Questionnaire (ODQ).	no data
Schmidt et al., 2024, Germany	RCT	Intervention: 20 Follow-up: 16 (for the second follow-up, with LOCF used for missing data)	M = 33.9/6/14 (intervention)	Yes	approx. 5 months **OT** M = 21 ± 15 weeks **TNC** M = 20 ± 13 weeks	(1): OT (2): OT + topical nasal corticoid (TNC)	4 scents: Rose, Lemon, Eucalyptus, Cloves, 5 min, twice daily.	2 and 3 months	**Objective:** Sniffin' Sticks test (TDI).	**Interaction between time and group** d = 0.482; *p* > 0.05
Yaylaci et al., 2023, Turkey	Comparative study	Intervention: 51 Follow-up: 43	M = 37.5, Md = 38/19/24 (follow-up)	Yes	approx. 5.25 months **OT** M = 5.8 ± 3.4 months **Control group** M = 4.7 ± 3.3 months	(1) Classical olfactory training group: OT (2) Control group: patients who decided to wait for spontaneous recovery	4 scents: Rose, Lemon, Eucalyptus, Cloves, 3-5 min, twice daily.	3 months	**Objective**: Sniffin' Sticks test (TDI).	**Classical olfactory training group** d = 1.014; *p* < 0.001 **Control group** d = 0.224; *p* > 0.05

All studies were performed on patients with a chronic olfactory disorder persisting for at least 4 weeks. All participants in the study took part in olfactory training. In most studies (10 out of 14), the traditional treatment regimen proposed by Hummel et al. (2009) was applied. Four scents were used: rose, lemon, eucalyptus, and clove. In one of the articles (Bérubé et al., 2023), lemon was substituted with orange (due to supply chain issues during the COVID pandemic). In most studies, four scents were used. Only in the study by Pires et al. (2022), advanced olfactory training (AOT) was applied using eight essential oils. Olfactory training was performed twice a day (10 out of 14) or three times a day (4 out of 14). Olfactory training was conducted for a minimum of 1 month. In only two studies, the OT lasted < 8 weeks (Pires et al., 2022; Chung et al., 2023). In all the studies, objective and proven testing tools were used for the outcome measures analyzed, most commonly the Sniffin' Sticks test (8 out of 14). However, in two studies, only the Sniffin' Sticks identification test was used, and in one study, only the Sniffin' Sticks threshold test was utilized.

### 3.1 Olfactory training alone

Five out of fourteen studies assessed the effects of OT alone (Pires et al., 2022; Bérubé et al., 2023; Khan et al., 2023; Lechien et al., 2023; Yaylaci et al., 2023). Most of the studies among those reviewed here demonstrate that olfactory training alone improves olfactory function (Pires et al., 2022; Khan et al., 2023; Lechien et al., 2023; Yaylaci et al., 2023). Improvement in objective measures of olfactory dysfunction following olfactory training was observed in 4 studies (Pires et al., 2022; Khan et al., 2023; Lechien et al., 2023; Yaylaci et al., 2023). Bérubé et al. (2023) observed no effect of OT on objective olfactory test scores. The participants were divided into two groups: the OT group and the placebo group. The authors noted a significant improvement in subjective olfactory function (3.8 ± 1.9 to 5.4 ± 1.8, *p* = 0.002) and a reduction in the frequency of parosmia in the OT group (16 to 14), while the placebo group did not exhibit these effects. Khan et al. (2023), recommend caution in interpreting the results. In a randomized, single-blinded study, participants were assigned to one of five groups differing in the type of olfactory training (OT): (1) bimodal training with patient-preferred scents, (2) bimodal training with physician-assigned scents, (3) unimodal training with patient-preferred scents, (4) unimodal training with physician-assigned scents, and (5) control group. Although all intervention arms showed a higher proportion of responders (37.6%) compared to the control group (24%), with specific differences as follows: bimodal patient-preferred: 29% (4.4%−53.9%), bimodal physician-assigned: 16.7% (−7.4%−40.9%), unimodal patient-preferred: 6.2% (−16.9%−29.3%), and unimodal physician-assigned: 5.1% (−18.1%−28.4%), the authors caution that the imprecision of these estimates prevents drawing definitive conclusions. At the same time, 55% of participants reported a subjective improvement in olfactory function.

### 3.2 Adjuvant therapy and olfactory training

Nine out of fourteen studies assessed the combined effects of adjuvant therapy and olfactory training. Four studies examined the combination of corticosteroids and OT (Saussez et al., 2021; Schepens et al., 2022; de Sousa et al., 2023; Schmidt et al., 2024). Two studies investigated a mineral supplement containing Palmitoylethanolamide (PEA) and Luteolin alongside OT (Di Stadio et al., 2022; Cantone et al., 2024). Two studies explored the use of antioxidants such as alpha-lipoic acid (ALA) with OT (Cantone et al., 2024; Figueiredo et al., 2024). Another study assessed the effects of oral vitamin A with OT (Chung et al., 2023), and one more looked into the benefits of cerebrolysin in conjunction with OT (Hamed and Ahmed, 2023).

#### 3.2.1 Corticosteroids and olfactory training

The results of studies on the effects of combining corticosteroids with olfactory training are conflicting. While studies by Saussez et al. (2021) and de Sousa et al. (2023) indicate a significant initial improvement in olfactory function with combined therapy, others, such as those by Schepens et al. (2022) and Schmidt et al. (2024), found no substantial differences between groups receiving corticosteroids with OT and those undergoing OT alone. These findings suggest that the benefits of adjunctive therapies may also depend on the specific treatment used.

Studies by Saussez et al. (2021) and de Sousa et al. (2023) demonstrate that combining olfactory training with additional therapies results in a greater initial improvement in olfactory function compared to OT alone. In the non-randomized study conducted by de Sousa et al. (2023), 47 participants were divided into one of four groups based on their nasal status (depending on endoscopic findings) and treatment preferences. The first group received 50 μg of mometasone nasally, twice daily, complemented by a twice-daily multivitamin B supplement and OT. The second group received the same dosage of mometasone without any vitamin supplements, plus OT. The third group applied an intranasal solution containing Vitamin A and Vitamin E twice daily, along with OT. The fourth group engaged in olfactory training alone, with no additional adjuvant therapies. At the 3-month follow-up, all groups demonstrated notable enhancements in olfactory thresholds. However, the average improvement for olfactory training alone was lower at 2.9, compared to 4.3 for the combination of olfactory training with adjuvant therapies. No significant differences were noted in the outcomes between specific adjuvant subgroups.

In a non-randomized European multicenter study, 152 individuals participated (Saussez et al., 2021). The participants were divided into three groups. Group 1 (OC + OT group) comprised patients who underwent a 10-day regimen of oral corticosteroids (methylprednisolone at 0.5 mg/kg/day) combined with olfactory training. Group 2 (NC + OT group) consisted of patients who received a month-long treatment with nasal corticosteroids (mometasone furoate spray, two sprays in each nostril once daily), also paired with OT. Group 3 (OT) included patients who were provided with olfactory training alone. At the 1-month post-treatment mark, the median olfactory score improvement for the OC + OT group (6, IQR 4) was significantly greater compared to the NC + OT group (3, IQR 5.25; *p* < 0.001) and the OT group (4, IQR 3; *p* < 0.001). No significant differences were observed in score improvements between the NC + OT and OT groups (*p* = 0.999). At 2 months post-treatment, there were no significant differences in median olfactory score improvements among the groups. Similar results were observed regarding the proportion of normosmics. At 2 months, there were no significant differences in the proportion of normosmics among the groups.

Both Schepens et al. (2022) and Schmidt et al. (2024) studies found no significant differences in olfactory improvements between groups receiving corticosteroids with OT and those receiving OT alone. No difference in olfactory function between the treatment group, which received oral prednisolone 40 mg once daily for 10 days along with OT, and the placebo group, which received a matching placebo and OT, was observed in a randomized, double-blind, placebo-controlled trial conducted in the Netherlands (Schepens et al., 2022). The median TDI score was 26.8 (IQR 23.6–29.3) in the placebo group and 28.8 (IQR 24.0–30.9) in the prednisolone group, resulting in a median difference of −1.5 (−3.0 to 0.25, *p* = 0.01). However, both groups experienced an improvement in olfactory function after 12 weeks. No difference in olfactory function between the treatment group, which received topical nasal corticosteroids (TNC) with OT, and the group that received only OT, was observed in a randomized controlled trial (RCT) in Germany (Schmidt et al., 2024). Both groups (OT alone and OT with additional therapy) demonstrated a significant overall improvement in olfactory ability over time. The group receiving only OT experienced a more rapid initial improvement compared to the combination therapy group.

#### 3.2.2 Palmitoylethanolamide and Luteolin and olfactory training

For our review, we included two randomized studies from the Italian center led by Di Stadio et al. (2022) and Cantone et al. (2024), involving a total of 305 patients. In each study, combining CoUltraPEALut supplementation with olfactory training resulted in greater improvements in olfactory function compared to olfactory training alone. Di Stadio et al. (2022) conducted a multicenter, double-blinded, randomized, placebo-controlled clinical study. The participants were randomized to receive daily treatment with ultra-micronized PEA-LUT 770 mg + OT (intervention group) or OT with a placebo (control group). The results showed that 92% of patients in the intervention group improved, compared to 42% in the control group. The mean TDI score increased from 20.6 ± 7.9 at T0 to 29.8 ± 7.5 at T3 (*p* = 0.0001) in the intervention group. Mean TDI scores in the control group did not significantly change from T0 (18.2 ± 7.9) to T3 (19.5 ± 7.3) (*p* = 0.4). Specifically, 56% of patients in the intervention group recovered to a normal TDI (>31), compared to only 10% in the control group. Similar results were achieved in another randomized study conducted by the Italian team (Cantone et al., 2024). In this study, treatment with umPEALUT+OT was associated with higher recovery of TDI scores. The combination of umPEALUT+ALA+OT (19.6 ± 6.29 to 27.5 ± 2.5) improved parosmia but had less effect on TDI scores compared to umPEALUT+OT (18.6 ± 10.45 to 29.7 ± 7.5). However, ALA+OT (19.3 ± 5.6 to 21.7 ± 4.3) or OT alone (26.9 ± 5.3 to 27.7 ± 5) provided little benefit. However, it should be noted that the control group started with a significantly higher baseline TDI score (26.9 ± 5.3) compared to the other groups. The baseline comparison revealed statistically significant differences between the control group and the umPEALUT group (*p* < 0.05), the control group and the ALA group (*p* < 0.01), as well as between the control group and the umPEALUT + ALA group (*p* < 0.01).

#### 3.2.3 Other adjuvants

In our systematic review, we included studies that examined the effects of various adjuvants, such as alpha-lipoic acid, cerebrolysin, and Vitamin A, in combination with olfactory training for treating COVID-19-related olfactory dysfunction (Chung et al., 2023; Hamed and Ahmed, 2023; Cantone et al., 2024; Figueiredo et al., 2024). The use of ALA and OT for treating COVID-19-related olfactory dysfunction remains controversial and shows limited effectiveness (Cantone et al., 2024; Figueiredo et al., 2024), while cerebrolysin demonstrated rapid and promising results (Hamed and Ahmed, 2023), and combination therapy with Vitamin A and OT significantly improved olfactory function and neural activity (Chung et al., 2023).

Hummel et al. (2002) proposed combining ALA with OT to aid the recovery of olfactory functions lost due to viral infections of the upper respiratory tract. However, its effectiveness in conjunction with OT in treating COVID-19-related olfactory dysfunction remains controversial. Cantone et al. (2024) demonstrated that ALA+OT provided little therapeutic benefit for patients with chronic olfactory disorders. Also, in a randomized study, it was found that alpha-lipoic acid as an adjuvant to olfactory training did not show significant benefits for recovering smell loss due to COVID-19 (Figueiredo et al., 2024). Both groups, the intervention group (alpha-lipoic acid + OT) and the placebo group (starch pills + OT), achieved improvement in both subjective and objective measures of olfaction 12 weeks after treatment. However, there were no significant differences between the groups in CCCRC score (*p* = 0.63), mean olfactory threshold scores (*p* = 0.50), identification scores (*p* = 0.96), and VAS scores (*p* = 0.97). After 12 weeks, the frequency of anosmia reduced to 2% in the intervention group and to 7.8% in the placebo group. Additionally, 16.8% of intervention subjects and 15.7% of patients in the comparison group reached normosmia.

Cerebrolysin demonstrated a fast, promising, and consistent effect in the treatment of olfactory dysfunction (Hamed and Ahmed, 2023). In a prospective randomized trial, patients were assigned to one of two groups. Group 1 received cerebrolysin [5 ml/d (IM), 5 d/week] and practiced olfactory and gustatory training, while Group 2 practiced olfactory and gustatory training only. Recovery (normosmic) was complete in 61.5% of patients receiving cerebrolysin therapy and partial (hyposmic instead of anosmic) in 17%. In contrast, there was no recovery in the group that only practiced olfactory and gustatory training.

In a randomized controlled trial, three groups were compared: the combination group received oral Vitamin A and OT, the standard care group received only OT, and the control group was under clinical observation (Chung et al., 2023). The results showed that the combination therapy significantly improved olfactory function (increase in BTT scores, *p* < 0.001, MD = 4.4, 95% CI 1.7 to 7.2) compared to OT alone. Additionally, increased neural activity in the olfactory functional network and higher NAA/Cr ratios were observed, indicating the presence of viable neurons within the olfactory system.

### 3.3 Evaluating different protocols in olfactory training

In the majority of the reviewed research, the traditional olfactory training regimen proposed by Hummel et al. (2009) was applied. Bimodal patient-preferred training showed promising results (Khan et al., 2023), but further studies are needed to confirm its effectiveness. The importance of adherence to the training protocol was also emphasized (Lechien et al., 2023). Furthermore, the duration of OT plays a crucial role, with most studies indicating that a period of at least 3 months is necessary for meaningful improvement in olfactory function (Schepens et al., 2022; Khan et al., 2023; Lechien et al., 2023; Yaylaci et al., 2023; Figueiredo et al., 2024; Schmidt et al., 2024).

In most studies (10 out of 14), the traditional treatment regimen proposed by Hummel et al. (2009) was applied. An alternative approach was used by Pires et al. (2022). In a multicenter randomized clinical trial, patients were divided into two groups: one group received Classical Olfactory Training (COT) using 4 essential oils, while the other group underwent Advanced Olfactory Training (AOT) using 8 essential oils. When comparing the two treatment groups (COT and AOT), there were no significant differences in UPSIT scores or olfaction VAS scores.

Khan et al. (2023) introduced two modifications in olfactory training (OT). Participants randomized to the patient-preferred groups could select 4 scents from a total of 24 options, covering 6 odor categories—fruity, citrus, earthy, floral, mint, and spice. The authors also implemented bimodal training (visual-olfactory), where participants were shown digital images corresponding to the essential oil they were smelling. This trial did not show any differences between the intervention groups. There was no significant difference in the change in UPSIT scores from pre-intervention to post-intervention between participants assigned to patient-preferred OT and those assigned to physician-assigned olfactory training (marginal mean difference, 0.73; 95% CI, −1.10 to 2.56). Similarly, no significant difference was observed between participants assigned to visual training and those without the visual component (marginal mean difference, 1.10; 95% CI, −2.92 to 0.74). Interestingly, when considering the percentage of participants who experienced a clinically meaningful improvement in UPSIT scores, the highest proportion of improvement was observed in the bimodal groups, specifically in the bimodal patient-preferred group.

Non-traditional olfactory training was also implemented by Hamed and Ahmed (2023). The training involved the use of four strong odorants or scents, such as lemon, curry powder, lavender, pungent herbs, spices like cloves, and various essential oils. The duration and number of daily olfactory training sessions were similar to the classic procedure. It was shown that there was no improvement in olfactory function in the group that only practiced olfactory and gustatory training. A different OT procedure was also implemented by Saussez et al. (2021). The authors utilized a procedure with more than three daily odors (e.g., coffee, perfume, essential oils) for each OT session. The authors determined that after 2 months of training, olfactory training alone provided benefits similar to those observed in groups with therapeutic intervention.

Adherence to the therapeutic protocol is crucial. Participants undergoing OT were divided into two groups: full adherence to OT and non-adherence to OT (Lechien et al., 2023). The researchers showed that adherence to an olfactory training protocol was associated with better mid-term improvement of psychophysical scores. Patients who did not adhere to OT showed significant improvement in psychophysical scores from baseline (6.9 ± 3.0) to 6 months post-infection (10.1 ± 3.7, *p* < 0.01). However, after this time, there was no further improvement in identification evaluations at 12 months (11.3 ± 4.6, *p* = NS) and 18 months (12.9 ± 4.1, *p* = NS). In the OT group, scores significantly improved from baseline (7.2 ± 2.7) not only to 6 months post-infection (10.2 ± 4.1, *p* < 0.001) but also to 12 months post-infection (11.8 ± 3.3, *p* < 0.05), suggesting more sustained and prolonged benefits from OT. Nevertheless, in both groups—those not adhering to OT (12.9 ± 4.1) and those adhering to OT (14.1 ± 2.4)—most patients achieved normosmia.

The duration of the OT is also important. Hummel et al. (2017) recommend 8 weeks of training to validate the effect. In only two studies, the OT lasted < 8 weeks (Pires et al., 2022; Chung et al., 2023). In the majority of reviewed studies, an improvement in olfactory function was observed due to 3 months or more of OT (Schepens et al., 2022; Khan et al., 2023; Lechien et al., 2023; Yaylaci et al., 2023; Figueiredo et al., 2024; Schmidt et al., 2024). In one study, more significant improvements were noted when combined therapies were used (de Sousa et al., 2023). In three studies, no improvement was observed in the group with only 3 months or more of OT (Di Stadio et al., 2022; Hamed and Ahmed, 2023; Cantone et al., 2024). However, in the study by Cantone et al., a ceiling effect might have occurred (the higher baseline TDI score in the control group). In 1-month olfactory training, no significant improvement in olfactory scores was noted (Saussez et al., 2021; Chung et al., 2023).

## 4 Discussion

Olfactory training is a non-pharmacological treatment for olfactory loss, aimed at enhancing smell function through neural rearrangement and reorganization. A meta-analysis by Asvapoositkul et al. (2023) highlighted OT as the most recommended approach for managing post-COVID-19 olfactory dysfunction. Similarly, a meta-analysis by Kattar et al. (2021) reported significant improvements in post-viral olfactory dysfunction with OT. However, the long-term effectiveness of OT for treating persistent olfactory disorders associated with long COVID is still not well understood.

Our review found that olfactory training was effective in treating chronic olfactory disorders after COVID-19 (Bérubé et al., 2023; de Sousa et al., 2023; Khan et al., 2023; Lechien et al., 2023; Figueiredo et al., 2024; Schmidt et al., 2024). Most studies indicate a positive impact of olfactory training on olfactory dysfunction after COVID-19, both in objective measures and in subjective perceptions of patients. Only three studies showed the ineffectiveness of OT (Di Stadio et al., 2022; Hamed and Ahmed, 2023; Cantone et al., 2024). The authors suggest that olfactory training only combined with adjuvants can lead to significant improvement in olfactory function in patients after COVID-19. In the studies by Di Stadio et al. (2022) only 10% of patients in the control group (OT alone) recovered to a normal TDI. Similarly, Hamed and Ahmed (2023) emphasize that there was no recovery in the group receiving only OT. Cantone et al. (2024) showed little OT benefits, but the control group started with a significantly higher baseline TDI score (26.9 ± 5.3) compared to the other groups. The higher baseline TDI score in the control group could have influenced the overall results by making it more challenging to observe significant improvements in this group compared to the other groups. This initial advantage may have led to a ceiling effect, whereby the potential for further improvement is limited due to already high starting scores. It should be noted, however, that the score obtained by the control group, according to the TDI score, should be defined as hyposmia (similarly to the baseline scores in other study groups). Nevertheless, despite the interventions, none of the groups in the study by Cantone et al. (2024), including those receiving OT combined with adjuvants, achieved normosmia. The greatest improvement was seen with the combination of umPEALUT+OT (18.6 ± 10.45 to 29.7 ± 7.5), but the results still fell within the range of hyposmia. Given that the control group had a higher baseline score, the actual effect of OT therapy alone may be harder to estimate in this group.

Our review revealed that the majority of studies utilized traditional training, which included four well-known scents: rose, lemon, eucalyptus, and cloves, following the procedure proposed by Hummel et al. (2009). According to our analysis, both traditional and modified OT have shown significant improvement in olfaction (Saussez et al., 2021; Pires et al., 2022; Khan et al., 2023), although the superiority of either one has not been clearly established. The intensification of OT (using 8 essential oils) is not more effective than classical training over a 4-week period (Pires et al., 2022). Despite the lack of significant differences, the highest percentage of participants who experienced a clinically meaningful improvement in objective scores was observed in the bimodal training group, particularly among those who engaged in patient-preferred bimodal training (Khan et al., 2023). Bimodal training involves the use of visual and olfactory stimuli together, whereby participants are shown digital images corresponding to the essential oil they are smelling. It is possible that this approach, combined with patient-preferred scents, enhances the effectiveness of the training by engaging multiple senses and increasing participant motivation. However, more research is needed to confirm this hypothesis and to determine the long-term benefits and optimal implementation strategies for bimodal training.

Adherence to the protocol is crucial for the effectiveness of OT. The study by Lechien et al. (2023) showed that patients who strictly followed the protocol achieved better results in the mid-term compared to those who did not. However, not all studies verified whether patients adhered to the protocol. Additionally, the number of weeks of OT is significant for its effectiveness. Hummel et al. (2017) suggest an 8-week training period to confirm the effect. There are reports indicating that olfactory training is effective when conducted for a duration of 3 months or more (Pieniak et al., 2022). Most reviewed studies (Schepens et al., 2022; Khan et al., 2023; Lechien et al., 2023; Yaylaci et al., 2023; Figueiredo et al., 2024; Schmidt et al., 2024) observed an improvement in olfactory function after 3 months or longer of OT. A 1-month training period was found to be insufficient (Saussez et al., 2021; Chung et al., 2023). In the study by Saussez et al. (2021), 1-month olfactory training did not lead to improvement. It was only after extending OT to 2 months that the expected enhancement in olfactory function was achieved. The 1-month improvement was observed only when OT was combined with an adjuvant. The addition of oral corticosteroids to the treatment accelerated the recovery of olfactory function, with noticeable improvements by the 1-month mark.

In our review, we also examined the effect of OT combined with an adjuvant. We suggest that olfactory training combined with adjuvants can lead to significant improvements in chronic olfactory function in patients after COVID-19; however, the results vary depending on the adjuvants used and the specific study configuration. Our systematic review highlighted the significant benefits of CoUltraPEALut (Di Stadio et al., 2022; Cantone et al., 2024), Cerebrolysin (Hamed and Ahmed, 2023) and oral Vitamin A (Chung et al., 2023) treatments with OT for chronic olfactory dysfunction following COVID-19. Alpha-lipoic acid (ALA) therapy: despite some positive effects, it is not more effective than OT alone in treating olfactory disorders after COVID-19 (Cantone et al., 2024; Figueiredo et al., 2024).

For our review, we decided to include two articles from studies conducted by the Italian team on the effectiveness of CoUltraPEALut in treating chronic olfactory dysfunction after COVID-19. We were unable to find studies by other teams. Both studies by Di Stadio et al. (2022) and Cantone et al. (2024) demonstrated significant improvements in olfactory function as a result of combined treatment with PEA-LUT and olfactory training. The authors suggest that PEA-LUT with olfactory training resulted in greater recovery of smell than olfactory training alone. Also, the systematic review and meta-analysis by Capra et al. (2023) underscored the significant benefits of CoUltraPEALut treatment for olfactory dysfunction following COVID-19. However, the analysis was limited to just five studies involving a total of 441 subjects. Additionally, all the studies included in the review were conducted by the Italian team led by Arianna Di Stadio. PEA (Palmitoylethanolamide) and LUT (Luteolin) are compounds studied for their therapeutic effects. PEA is a natural fatty acid amide with anti-inflammatory and pain-relieving properties. Luteolin is a flavonoid found in plants and fruits, known for its anti-inflammatory, anti-allergic, and anti-tumor activities. It also functions as an antioxidant and pro-oxidant (Imran et al., 2019). Together, PEA and LUT are researched for their combined benefits in treating conditions like anosmia due to their anti-inflammatory and neuroprotective effects. Promising results have also been achieved with the Cerebrolysin and OT therapy proposed by Hamed and Ahmed (2023) as well as oral Vitamin A with OT (Chung et al., 2023). Cerebrolysin, a drug with neurotrophic and neuroprotective properties, is primarily approved for treating dementia, acute ischemic stroke, cognitive impairment (Brainin, 2018; Cui et al., 2019). It is widely used in Post-Soviet states, Eastern Europe, China, Asia, Russia, South Korea, and Arab countries (Hamed and Ahmed, 2023).

There are conflicting results regarding corticosteroid therapy: while some studies indicate potential benefits (Saussez et al., 2021; de Sousa et al., 2023) of including corticosteroids in the treatment of olfactory disorders after COVID-19, other studies do not confirm these effects (Schepens et al., 2022; Schmidt et al., 2024). In the studies by Schepens et al. (2022) and Schmidt et al. (2024), an improvement was indeed noted as a result of combined corticosteroid and OT therapy. However, this effect was the same as that observed with OT therapy alone. What's more, Schepens et al. (2022) do not recommend prescribing prednisolone for patients with chronic olfactory disorders after COVID-19. Although the researchers emphasize that the lack of therapeutic effect may be due to the late initiation of the medication, as treatment should ideally start within 72 h after the onset of symptoms. Nevertheless, early treatment with prednisolone can inhibit the immune response against COVID-19, potentially leading to a prolonged infection. Saussez et al. (2021) suggest that oral corticosteroids combined with OT are more effective than nasal corticosteroids, especially in the short term. However, other studies (Schepens et al., 2022; de Sousa et al., 2023; Schmidt et al., 2024) do not unequivocally confirm these results.

The study has several limitations. First, the relatively young age of the patients in our review may have contributed to their better response to OT therapy, as younger individuals typically exhibit lower levels of neuroinflammation compared to the elderly. Age-related olfactory loss is less prevalent in younger age groups, and younger individuals generally possess a greater capacity for neuroregeneration (Welge-Lüssen, 2009; Fatuzzo et al., 2023). Secondly, we found only 14 relevant studies, resulting in a relatively small sample size. In the case of certain adjuvants, our findings are based only a single study. It is important to note that the promising results observed with cerebrolysin (Hamed and Ahmed, 2023) and the combination therapy of Vitamin A and OT (Chung et al., 2023) are based on single studies, highlighting the need for further research.

## 5 Conclusion and future perspectives

Our study provided evidence of the effectiveness of olfactory training in improving olfactory function in cases of persistent smell disorders related to COVID-19. The results concerning therapy combined with OT are also promising. A combination of olfactory training with adjuvants, such as CoUltraPEALut, Cerebrolysin, and oral Vitamin A, may be associated with improvements in post-COVID-19 olfactory function; however, this requires further analysis to confirm its effectiveness. Adherence to the OT protocol and longer durations of OT, typically 3 months or more, significantly influence treatment outcomes. Despite the encouraging evidence, it is essential to emphasize the need for further large-scale clinical trials to validate these positive outcomes and gain a deeper understanding of the mechanisms by which OT promotes olfactory recovery both alone and in combination with adjuvants. Future research should investigate OT in combination with other therapeutic modalities to better understand its potential synergistic effects. Further studies involving patients from various age groups, particularly seniors, are also necessary.

## Data Availability

The original contributions presented in the study are included in the article/supplementary material, further inquiries can be directed to the corresponding author.

## References

[B1] Asvapoositkul V. Samuthpongtorn J. Aeumjaturapat S. Snidvongs K. Chusakul S. Seresirikachorn K. . (2023). Therapeutic options of post-COVID-19 related olfactory dysfunction: a systematic review and meta-analysis. Rhinology 61, 2–11. 10.4193/Rhin22.221 36173148

[B2] Bérubé S. Demers C. Bussière N. Cloutier F. Pek V. Chen A. . (2023). Olfactory training impacts olfactory dysfunction induced by COVID-19: a pilot study. ORL J. Otorhinolaryngol. Relat. Spec. 85, 57–66. 10.1159/000528188 36529118 PMC9843729

[B3] Boscolo-Rizzo P. Hummel T. Hopkins C. Dibattista M. Menini A. Spinato G. . (2021). High prevalence of long-term olfactory, gustatory, and chemesthesis dysfunction in post-COVID-19 patients: a matched case-control study with one-year follow-up using a comprehensive psychophysical evaluation. Rhinology 59, 517–527. 10.4193/Rhin21.249 34553706

[B4] Brainin M. (2018). Cerebrolysin: a multi-target drug for recovery after stroke. Expert Rev. Neurother. 18, 681–687. 10.1080/14737175.2018.1500459 30004268

[B5] Cain W. S. Gent J. F. Goodspeed R. B. Leonard G. (1988). Evaluation of olfactory dysfunction in the Connecticut Chemosensory Clinical Research Center. Laryngoscope 98, 83–88. 10.1288/00005537-198801000-00017 3336267

[B6] Cantone E. D'Ascanio L. De Luca P. Roccamatisi D. La La Mantia I. Brenner M. J. . (2024). Persistent COVID-19 parosmia and olfactory loss post olfactory training: randomized clinical trial comparing central and peripheral-acting therapeutics. Eur. Arch. Otorhinolaryngol. 281, 3671–3678. 10.1007/s00405-024-08548-6 38492007 PMC11211159

[B7] Capra A. P. Ardizzone A. Crupi L. Calapai F. Campolo M. Cuzzocrea S. . (2023). Efficacy of palmitoylethanolamide and luteolin association on post-covid olfactory dysfunction: a systematic review and meta-analysis of clinical studies. Biomedicines 11:2189. 10.3390/biomedicines11082189 37626685 PMC10452638

[B8] Chung T. W. H. Zhang H. Wong F. K. C. Sridhar S. Lee T. M. C. Leung G. K. K. . (2023). A pilot study of short-course oral vitamin A and aerosolised diffuser olfactory training for the treatment of smell loss in long COVID. Brain Sci. 13:1014. 10.3390/brainsci13071014 37508945 PMC10377650

[B9] Courtiol E. Wilson D. A. (2017). The olfactory mosaic: bringing an olfactory network together for odor perception. Perception 46, 320–332. 10.1177/0301006616663216 27687814 PMC5362339

[B10] Cui S. Chen N. Yang M. Guo J. Zhou M. Zhu C. . (2019). Cerebrolysin for vascular dementia. Cochr. Datab. System. Rev. 2019:CD008900. 10.1002/14651858.CD008900.pub3 31710397 PMC6844361

[B11] D'Ascanio L. Vitelli F. Cingolani C. Maranzano M. Brenner M. J. Di Stadio A. (2021). Randomized clinical trial “olfactory dysfunction after COVID-19: olfactory rehabilitation therapy vs. intervention treatment with palmitoylethanolamide and luteolin”: Preliminary results. Eur. Rev. Med. Pharmacol. Sci. 25, 4156–4162. 10.26355/eurrev_202106_26059 34156697

[B12] de Sousa F. A. de Machado A. S. Costa J. C. da Silva A. C. Pinto A. N. Coutinho M. B. . (2023). Tailored approach for persistent olfactory dysfunction after SARS-CoV-2 infection: a pilot study. Ann. Otol. Rhinol. Laryngol. 132, 657–666. 10.1177/00034894221111093 35822286

[B13] Denis F. Septans A.-L. Periers L. Maillard J.-M. Legoff F. Gurden H. . (2021). Olfactory training and visual stimulation assisted by a web application for patients with persistent olfactory dysfunction after SARS-CoV-2 infection: observational study. J. Med. Internet Res. 23:e29583. 10.2196/29583 34003765 PMC8163493

[B14] Di Stadio A. D'Ascanio L. Vaira L. A. Cantone E. De Luca P. Cingolani C. . (2022). Ultramicronized palmitoylethanolamide and luteolin supplement combined with olfactory training to treat post-COVID-19 olfactory impairment: a multi-center double-blinded randomized placebo-controlled clinical trial. Curr. Neuropharmacol. 20, 2001–2012. 10.2174/1570159X20666220420113513 35450527 PMC9886808

[B15] Doty R. L. Shaman P. Kimmelman C. P. Dann M. S. (1984). University of Pennsylvania Smell Identification Test: a rapid quantitative olfactory function test for the clinic. Laryngoscope 94, 176–178. 10.1288/00005537-198402000-00004 6694486

[B16] Fatuzzo I. Niccolini G. F. Zoccali F. Cavalcanti L. Bellizzi M. G. Riccardi G. . (2023). Neurons, nose, and neurodegenerative diseases: olfactory function and cognitive impairment. Int. J. Mol. Sci. 24, 2117. 10.3390/ijms24032117 36768440 PMC9916823

[B17] Ferreli F. Gaino F. Russo E. Di Bari M. Rossi V. De Virgilio A. . (2022). Long-term olfactory dysfunction in COVID-19 patients: 18-month follow-up study. Int. Forum Allergy Rhinol. 12, 1078–1080. 10.1002/alr.22990 35199476 PMC9082048

[B18] Figueiredo L. P. Paim P. V. D. S. L. Cerqueira-Silva T. Barreto C. C. Lessa M. M. (2024). Alpha-lipoic acid does not improve olfactory training results in olfactory loss due to COVID-19: a double-blind randomized trial. Braz. J. Otorhinolaryngol. 90:101356. 10.1016/j.bjorl.2023.101356 37944311 PMC10665681

[B19] Fjaeldstad A. W. Ovesen T. Stankevice D. Ovesen T. (2023). Olfactory training in long COVID-19 patients with lasting symptoms including olfactory dysfunction. Dan. Med. J. 70:A09220568. 36896723

[B20] Hamed S. A. Ahmed M. A. A.-R. (2023). The effectiveness of cerebrolysin, a multi-modal neurotrophic factor, for treatment of post-covid-19 persistent olfactory, gustatory and trigeminal chemosensory dysfunctions: a randomized clinical trial. Expert. Rev. Clin. Pharmacol. 16, 1261–1276. 10.1080/17512433.2023.2282715 37950370

[B21] Hopkins C. Gillett S. Slack R. Lund V. J. Browne J. P. (2009). Psychometric validity of the 22-item Sinonasal Outcome Test. Clin. Otolaryngol. 34, 447–454. 10.1111/j.1749-4486.2009.01995.x 19793277

[B22] Hosseini K. Zare-Sadeghi A. Sadigh-Eteghad S. Mirsalehi M. Khezerloo D. (2020). Effects of olfactory training on resting-state effective connectivity in patients with posttraumatic olfactory dysfunction. Acta Neurobiol. Exp. 80, 381–388. 10.21307/ane-2020-035 33350991

[B23] Huang T. Wei Y. Wu D. (2021). Effects of olfactory training on posttraumatic olfactory dysfunction: a systematic review and meta-analysis. Am. J. Rhinol. Allergy 35, 640–647. 10.1002/alr.22758 33486898 PMC8358954

[B24] Hummel T. Heilmann S. Hüttenbriuk K.-B. (2002). Lipoic acid in the treatment of smell dysfunction following viral infection of the upper respiratory tract. Laryngoscope 112, 2076–2080. 10.1097/00005537-200211000-00031 12439184

[B25] Hummel T. Rissom K. Reden J. Hähner A. Weidenbecher M. Hüttenbrink K.-B. (2009). Effects of olfactory training in patients with olfactory loss. Laryngoscope 119, 496–499. 10.1002/lary.20101 19235739

[B26] Hummel T. Sekinger B. Wolf S. R. Pauli E. Kobal G. (1997). ‘Sniffin' sticks': olfactory performance assessed by the combined testing of odor identification, odor discrimination and olfactory threshold. Chem. Senses 22, 39–52. 10.1093/chemse/22.1.39 9056084

[B27] Hummel T. Whitcroft K. L. Andrews P. Altundag A. Cinghi C. Costanzo R. M. . (2017). Position paper on olfactory dysfunction. Rhinol. Suppl. 54, 1–30. 10.4193/Rhino16.248 29528615

[B28] Hwang S. H. Kim J.-S. Choi B. Y. Kim J. K. Kim B. G. (2022). Practical review of olfactory training and COVID-19. J. Rhinol. 65, 1–15. 10.18787/jr.2022.00407 PMC11524376 39664302

[B29] Hwang S. H. Kim S. W. Basurrah M. A. Kim D. H. (2023). The efficacy of olfactory training as a treatment for olfactory disorders caused by Coronavirus Disease-2019: a systematic review and meta-analysis. Am. J. Rhinol. Allergy 37, 495–501. 10.1177/19458924221150977 36635974

[B30] Imran M. Rauf A. Abu-Izneid T. Nadeem M. Shariati M. A. Khan I. A. . (2019). Luteolin, a flavonoid, as an anticancer agent: a review. Biomed. Pharmacother. 112:108612. 10.1016/j.biopha.2019.108612 30798142

[B31] Kattar N. Do T. M. Unis G. D. Migneron M. R. Thomas A. J. McCoul E. D. (2021). Olfactory training for postviral olfactory dysfunction: systematic review and meta-analysis. Otolaryngol. Head Neck Surg. 164, 244–254. 10.1177/0194599820943550 32660334

[B32] Khan A. M. Piccirillo J. Kallogjeri D. Piccirillo J. F. (2023). Efficacy of combined visual-olfactory training with patient-preferred scents as treatment for patients with COVID-19 resultant olfactory loss: a randomized clinical trial. JAMA Otolaryngol. Head Neck Surg. 149, 141–149. 10.1001/jamaoto.2022.4112 36580304 PMC9857399

[B33] Kim B.-Y. Park J. Y. Kim E. J. Kim B. G. Kim S. W. Kim S. W. (2019). The neuroplastic effect of olfactory training to the recovery of olfactory system in mouse model. Int. Forum Allergy Rhinol. 9, 715–723. 10.1002/alr.22320 30793525 PMC6767412

[B34] Kollndorfer K. Fischmeister F. P. S. Kowalczyk K. Hoche E. Mueller C. A. Trattnig S. . (2015). Olfactory training induces changes in regional functional connectivity in patients with long-term smell loss. Neuroimage Clin. 9, 401–410. 10.1016/j.nicl.2015.09.004 26594622 PMC4590718

[B35] Le Bon S.-D. Konopnicki D. Pisarski N. Prunier L. Lechien J. R. Horoi M. (2021). Efficacy and safety of oral corticosteroids and olfactory training in the management of COVID-19-related loss of smell. Eur. Arch. Otorhinolaryngol. 278, 3113–3117. 10.1007/s00405-020-06520-8 33423106 PMC7796691

[B36] Lechien J. R. Vaira L. A. Saussez S. (2023). Effectiveness of olfactory training in COVID-19 patients with olfactory dysfunction: a prospective study. Eur. Arch. Otorhinolaryngol. 280, 1255–1263. 10.1007/s00405-022-07665-4 36153785 PMC9510568

[B37] Li W. Luxenberg E. Parrish T. Gottfried J. A. (2006). Learning to smell the roses: experience-dependent neural plasticity in human piriform and orbitofrontal cortices. Neuron 52, 1097–1108. 10.1016/j.neuron.2006.10.026 17178411 PMC1779760

[B38] Negoias S. Pietsch K. Hummel T. (2017). Changes in olfactory bulb volume following lateralized olfactory training. Brain Imaging Behav. 11, 998–1005. 10.1007/s11682-016-9567-9 27448159

[B39] Orji F. T. Akpeh J. O. Okolugbo N. E. (2023). Recovery patterns of COVID-19 related smell disorders: an analysis of the available evidence. Indian J. Otolaryngol. Head Neck Surg. 75, 4179–4189. 10.1007/s12070-023-04005-8 37974870 PMC10645952

[B40] Pekala K. Chandra R. K. Turner J. H. (2016). Efficacy of olfactory training in patients with olfactory loss: a systematic review and meta-analysis. Int. Forum Aller. Rhinol. 6, 299–307. 10.1002/alr.21669 26624966 PMC4783272

[B41] Pieniak M. Oleszkiewicz A. Avaro V. Calegari F. Hummel T. (2022). Olfactory training - Thirteen years of research reviewed. Neurosci. Biobehav. Rev. 141:104853. 10.1016/j.neubiorev.2022.104853 36064146

[B42] Pires Í. de A. T. Steffens S. T. Mocelin A. G. Shibukawa D. E. Leahy L. . (2022). Intensive olfactory training in post-COVID-19 patients: a multicenter randomized clinical trial. Am. J. Rhinol. Allergy 36, 780–787. 10.1177/19458924221113124 35866202 PMC9309586

[B43] Sarkis-Onofre R. Catalá-López F. Aromataris E. Lockwood C. (2021). How to properly use the PRISMA statement. Syst. Rev. 10:117. 10.1186/s13643-021-01671-z 33875004 PMC8056687

[B44] Saussez S. Vaira L. A. Chiesa-Estomba C. M. Le Bon S.-D. Horoi M. Deiana G. . (2021). Short-term efficacy and safety of oral and nasal corticosteroids in COVID-19 patients with olfactory dysfunction: a European multicenter study. Pathogens 10:698. 10.3390/pathogens10060698 34199734 PMC8228154

[B45] Schepens E. J. A. Blijleven E. E. Boek W. M. Boesveldt S. Stokroos R. J. Stegeman I. . (2022). Prednisolone does not improve olfactory function after COVID-19: a randomized, double-blind, placebo-controlled trial. BMC Med. 20:445. 10.1186/s12916-022-02625-5 36384737 PMC9667850

[B46] Schmidt F. Azar C. Goektas O. (2024). Treatment of olfactory disorders after SARS-CoV-2 virus infection. Ear. Nose Throat. J. 103, 48S-53S. 10.1177/01455613231168487 36976171 PMC10051008

[B47] Schwob J. E. (2002). Neural regeneration and the peripheral olfactory system. Anat. Rec. 269, 33–49. 10.1002/ar.10047 11891623

[B48] Sorokowska A. Drechsler E. Karwowski M. Hummel T. (2017). Effects of olfactory training: a meta-analysis. Rhinology 55, 17–26. 10.4193/Rhino16.195 28040824

[B49] Veronese N. Bonica R. Cotugno S. Tulone O. Camporeale M. Smith L. . (2022). Interventions for improving long COVID-19 symptomatology: a systematic review. Viruses 14:1863. 10.3390/v14091863 36146672 PMC9502379

[B50] Wang J.-Y. Pao J.-B. Lee C.-H. Wang J.-Y. Lee M.-C. Wu T.-T. (2023). Corticosteroids for COVID-19-induced olfactory dysfunction: A comprehensive systematic review and meta-analysis of randomized controlled trials. PLoS ONE 18:e0289172. 10.1371/journal.pone.0289172 38127940 PMC10734960

[B51] Welge-Lüssen A. (2009). Ageing, neurodegeneration, and olfactory and gustatory loss. B-ENT 5, 129–132. 20084814

[B52] WHO (2021). A clinical case definition of post COVID-19 condition by a Delphi consensus. World Health Organization. Available at: https://www.who.int/publications/i/item/WHO-2019-nCoV-Post_COVID-19_condition-Clinical_case_definition-2021.1 (accessed June 3, 2024).

[B53] Yaylaci A. Azak E. Önal A. Aktürk D. R. Karadenizli A. (2023). Effects of classical olfactory training in patients with COVID-19-related persistent loss of smell. Eur. Arch. Otorhinolaryngol. 280, 757–763. 10.1007/s00405-022-07570-w 35904631 PMC9335450

